# CircHIPK3 regulates the autophagy and apoptosis of hypoxia/reoxygenation-stimulated cardiomyocytes via the miR-20b-5p/ATG7 axis

**DOI:** 10.1038/s41420-021-00448-6

**Published:** 2021-04-06

**Authors:** Zhimei Qiu, Yan Wang, Weiwei Liu, Chaofu Li, Ranzun Zhao, Xianping Long, Jidong Rong, Wengweng Deng, Changyin Shen, Jinson Yuan, Wengming Chen, Bei Shi

**Affiliations:** 1 Department of Cardiology, Affiliated Hospital of Zunyi Medical University, Zunyi, 563000 China; 2 Department of Cardiology, The Second Affiliated Hospital of Zunyi Medical University, Zunyi, 563000 China

**Keywords:** Cell death, Non-coding RNAs

## Abstract

Autophagy and apoptosis are involved in myocardial ischemia/reperfusion (I/R) injury. Research indicates that circular RNA HIPK3 (circHIPK3) is crucial to cell autophagy and apoptosis in various cancer types. However, the role of circHIPK3 in the regulation of cardiomyocyte autophagy and apoptosis during I/R remains unknown. Our study aimed to examine the regulatory effect of circHIPK3 during myocardial I/R and investigate its mechanism in cardiomyocyte autophagy and apoptosis. Methods and results. The expression of circHIPK3 was upregulated during myocardial I/R injury and hypoxia/reoxygenation (H/R) injury of cardiomyocytes. To study the potential role of circHIPK3 in myocardial H/R injury, we performed gain-of-function and loss-of-function analyses of circHIPK3 in cardiomyocytes. Overexpression of circHIPK3 significantly promoted H/R-induced cardiomyocyte autophagy and cell injury (increased intracellular reactive oxygen species (ROS) and apoptosis) compared to those in the control group, while silencing of circHIPK3 showed the opposite effect. Further research found that circHIPK3 acted as an endogenous miR-20b-5p sponge to sequester and inhibit miR-20b-5p activity, resulting in increased ATG7 expression. In addition, miR-20b-5p inhibitors reversed the decrease in ATG7 induced by silencing circHIPK3. Conclusions. CircHIPK3 can accelerate cardiomyocyte autophagy and apoptosis during myocardial I/R injury through the miR-20b-5p/ATG7 axis. These data suggest that circHIPK3 may serve as a potential therapeutic target for I/R.

## Introduction

Ischemic heart disease is one of the leading causes of death worldwide^1^. Ischemia/reperfusion (I/R) refers to the reperfusion of the myocardium after a period of myocardial ischemia that leads to the aggravation of cardiac tissue damage, the occurrence of a malignant arrhythmia, and deterioration of cardiac function^2^. At present, research on I/R injury mainly focuses on oxidative stress, inflammation, calcium overload, autophagy, and apoptosis. Inflammation and oxidative stress can ultimately affect the structure and function of cardiomyocytes by regulating autophagy and apoptosis^3^. It is suggested that autophagy and apoptosis play an important role in myocardial I/R injury.

Autophagy is the process of transporting damaged, denatured, and aging long-lived proteins and organelles and other substrates to the lysosome for enzymatic digestion, reducing the substrates to their constituents for reuse by cells^4^. Normal levels of autophagy can protect cells from adverse external stimuli and play a benign self-protective role. However, excessive autophagy or insufficient levels of autophagy may disrupt the body’s balance and cause disease. Autophagy plays multiple roles in cardiovascular disease^5–7^. Apoptosis refers to autonomous programmed cell death controlled by genes. Apoptosis plays an irreplaceable role in maintaining the integrity and balance of a multicellular organism’s tissues or organs. The process of growth, development, survival, and death of multicellular organisms, is accompanied by the process of apoptosis. Cardiomyocyte apoptosis is the main cause of cell death in ischemia-reperfusion^8^.

Evidence shows that non-coding RNAs (ncRNAs), from microRNAs (miRNAs) to long noncoding RNAs (lncRNAs) or even circular RNAs (circRNAs), can mediate autophagy- and apoptosis-related gene transcription and posttranscriptional regulation by participating in autophagy and apoptosis regulatory networks^9^. CircRNAs are a novel class of ncRNAs with a covalent closed-loop structure and without a 5′ cap structure and a 3′ end polyA tail^10^. Recent studies have shown that circRNA plays an important role in cell proliferation, differentiation, autophagy, and apoptosis. For instance, autophagy-related circRNA ACR can attenuate myocardial I/R injury by regulating the Pink1/FAM65B pathway to inhibit autophagy^11^. Our previous research found that cardiomyocytes undergoing hypoxia preconditioning could release circHIPK3-rich exosomes, thereby regulating the proliferation and apoptosis of endothelial cells underneath oxidative stress damage^12,13^. Furthermore, recent studies have shown that circHIPK3 regulates autophagy in STK11 mutant lung cancer through miR-124-3p/STAT3/PRKAA/AMPKα signaling^14^. However, little is known about the role and mechanism of circHIPK3 in cardiomyocyte autophagy and apoptosis during I/R injury. In this study, we concentrated on the role of circHIPK3 in cardiomyocyte injury induced by hypoxia/reoxygenation (H/R). We found that circHIPK3 could promote H/R-induced autophagy and apoptosis. Importantly, we found that circHIPK3 could directly sponge miR-20b-5p to upregulate ATG7 expression and consequently enhance the I/R-induced autophagy and apoptosis of cardiomyocytes.

## Results

### Enhancement of autophagy activity concomitant with increased circHIPK3 expression after myocardial I/R injury and cardiomyocyte H/R injury

**Fig. 1:**
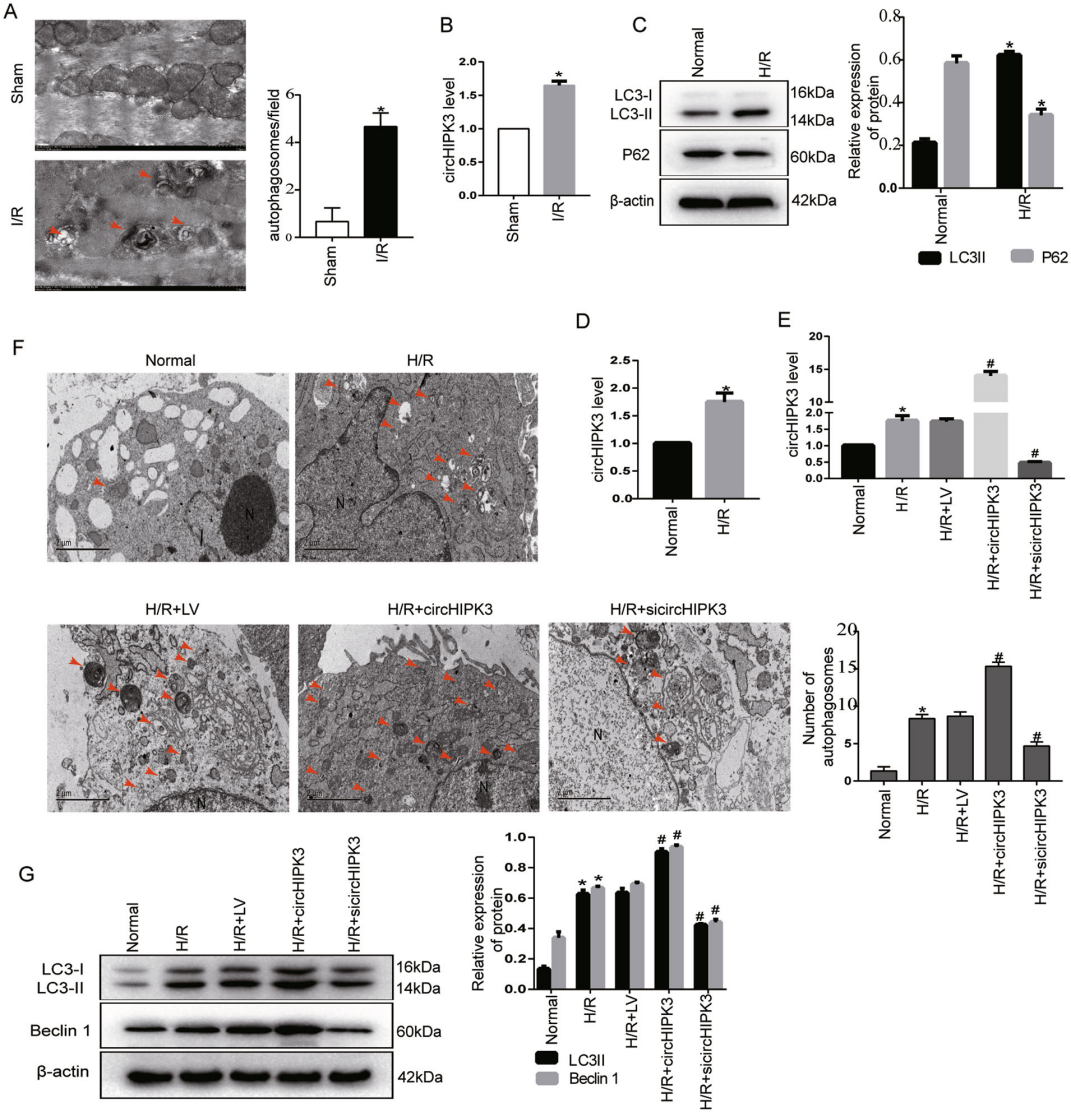
CircHIPK3 is upregulated concomitantly with accelerated autophagy in hypoxia/reoxygenation (H/R)-stimulated cardiomyocytes and ischemia/reperfusion (I/R)-stimulated myocardium. **A** Mice were subjected to 60 min of ischemia and 3 h of reperfusion. Representative transmission electron microscopy (TEM) images of hearts show the presence of autophagosomes (red arrow). Scale bar = 1 μm. **B** RT-qPCR analysis of circHIPK3 expression in mouse heart samples undergoing I/R or sham treatment. *n* = 3. **C** LC3-II and P62 expression were analyzed by western blotting in H/R (6 h hypoxia followed by 4 h reoxygenation)-stimulated cardiomyocytes. *n* = 3. **D** Increased expression of circHIPK3 in H/R-stimulated cardiomyocytes. *n* = 9. **E** Cardiomyocytes were transfected with negative control (LV), circHIPK3 overexpression vector, or circHIPK3 siRNA (si-circHIPK3) for 48 h. **F** Representative electron microscope analysis of autophagosomes in cardiomyocytes. Scale bar = 2 μm. **G** LC3-II and Beclin 1 expression were analyzed by western blotting in cardiomyocytes. *P < 0.05 compared with the sham or normal group. ^#^*P* < 0.05 compared with the H/R group.

To investigate the potential role of circHIPK3 in I/R myocardial autophagy, we detected the expression of circHIPK3 in mouse hearts with autophagy induced by I/R injury (Fig. 1A). The enhancement of autophagy activity after I/R injury was concomitant with increased circHIPK3 expression (Fig. 1B). Furthermore, we established H/R injury models in vitro to mimic I/R injury in vivo. We observed that H/R significantly induced cardiomyocyte autophagy (Fig. 1C). The conversion of LC3-I to LC3-II protein is considered a typical hallmark of autophagy^15^. Western blot analysis showed that the expression of endogenous LC3-II was markedly increased during H/R. P62 is an autophagy substrate^16^. As a reporter of autophagy activity, P62 degrades rapidly under H/R conditions. Notably, the qRT-PCR results showed that circHIPK3 was significantly upregulated in cardiomyocytes induced by H/R (Fig. 1D). To investigate the function of circHIPK3 in autophagy in H/R-injured cardiomyocytes, we carried out gain-of-function and loss-of-function analyses of circHIPK3 and explored its role in cardiomyocyte autophagy. Cardiomyocytes were transfected with circHIPK3 overexpression vector and si-circHIPK3. The transfection effects were confirmed by RT-qPCR (Fig. 1E). We detected morphological and quantitative changes in autophagic vacuoles (AVs) in cardiomyocytes by TEM. TEM results showed that circHIPK3 overexpression significantly increased the number of AVs compared to that in the H/R group, while circHIPK3 silencing (si-circHIPK3) decreased the number of AVs (Fig. 1F). Western blot analysis showed that the transition of LC3-I to LC3-II was markedly increased under circHIPK3 overexpression. Upon circHIPK3 silencing, decreased LC3-II expression inhibited the LC3-I/II conversion. Another autophagy-related protein Beclin1^17^ showed the same tendency (Fig. 1G). These results indicate that H/R-induced cardiomyocyte injury increases the expression of circHIPK3 and enhances cardiomyocyte autophagy.

### CircHIPK3 promoted H/R-induced apoptosis of cardiomyocytes

**Fig. 2:**
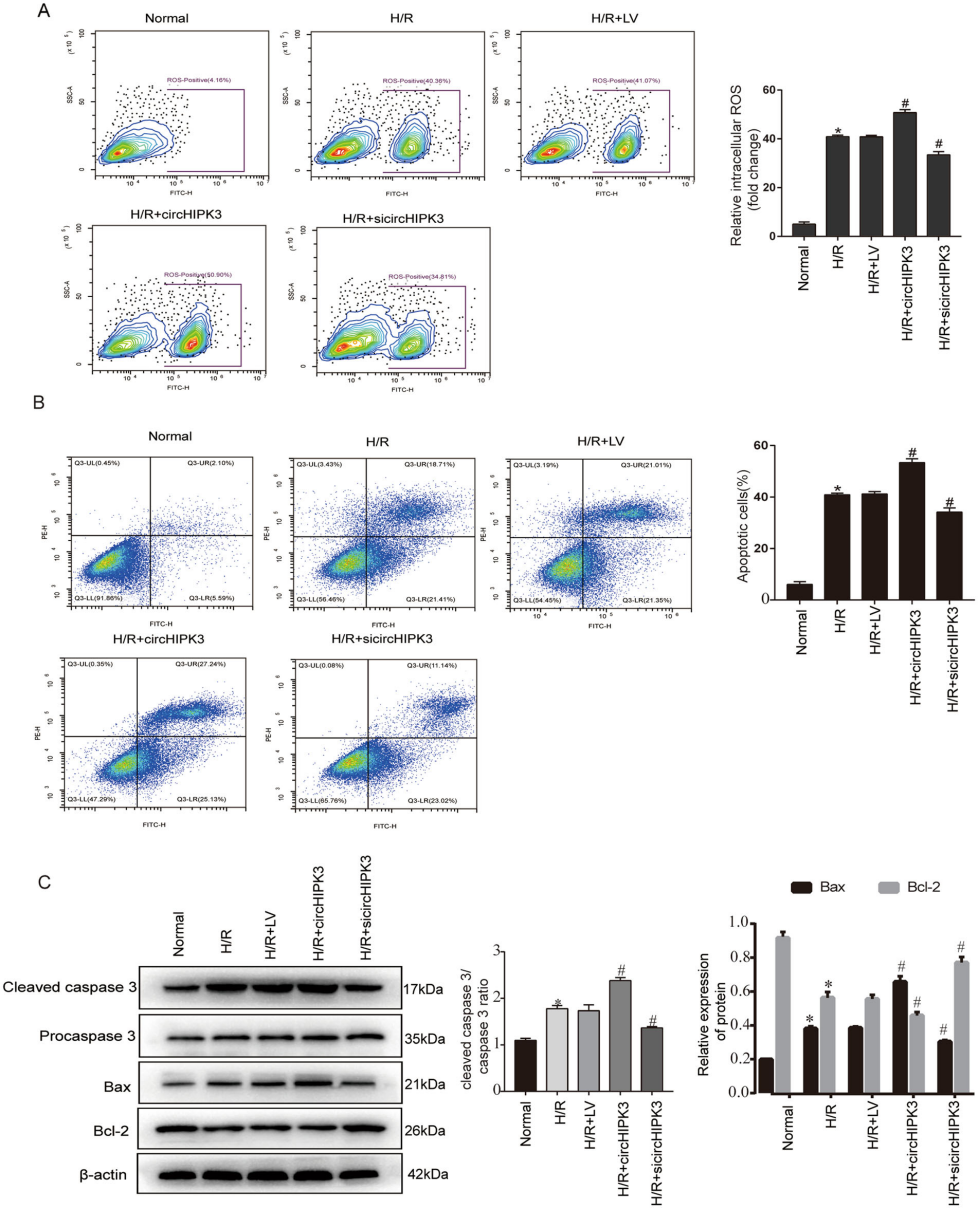
CircHIPK3 promotes H/R-induced cardiomyocyte apoptosis. **A** The intracellular ROS level was detected by flow cytometry. *n* = 3. **B** Annexin V-FITC/PI flow cytometry was used to evaluate the effect of circHIPK3 on cardiomyocyte apoptosis. *n* = 3. **C** Apoptosis-related proteins, including procaspase-3, cleaved caspase-3, Bax, and Bcl-2, were detected by western blotting. *n* = 3. *P < 0.05 compared with the normal group. ^#^*P* < 0.05 compared with the H/R group.

During the I/R process, when the ischemic myocardial tissue restores the supply of nutrients and oxygen, a large amount of ROS accumulates, which indicates that autophagy is significantly increased. The excessive increase in autophagy levels is usually accompanied by increased apoptosis of cardiomyocytes^18^. First, we observed that in cardiomyocytes under H/R conditions, overexpression of circHIPK3 significantly increased ROS accumulation, and circHIPK3 silencing reduced the production of ROS compared to that in the control group. (Fig. 2A). Next, we investigated whether circHIPK3 was involved in the regulation of H/R-induced apoptosis. Flow cytometry analysis showed that H/R-induced apoptosis was significantly increased after overexpression of circHIPK3, whereas circHIPK3 silencing inhibited H/R-induced apoptosis (Fig. 2B). In addition, overexpression of circHIPK3 noticeably increased the levels of the proapoptotic proteins cleaved caspase-3 and Bax and decreased the expression of the antiapoptotic protein Bcl-2 (Fig. 2C). Taken together, these findings indicated that circHIPK3 can increase H/R-induced apoptosis.

### CircHIPK3 acts as a miR-20b-5p sponge in cardiomyocytes

**Fig. 3:**
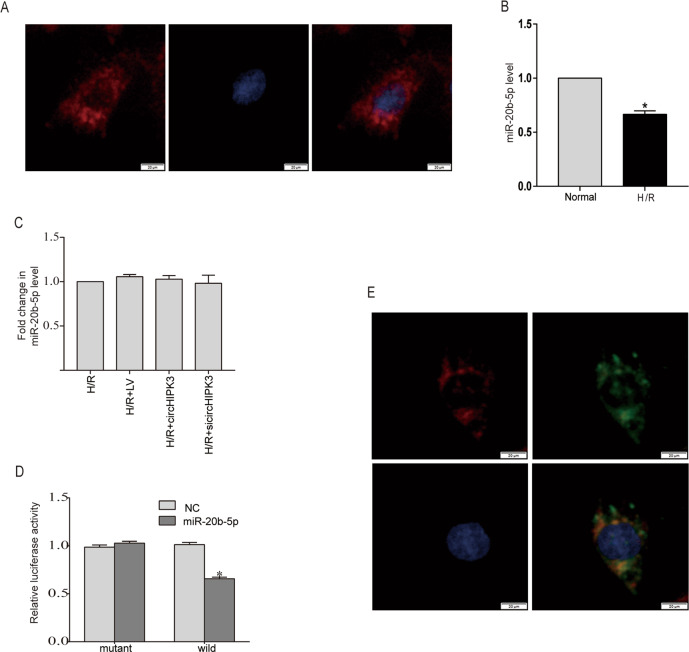
CircHIPK3 acts as a miR-20b-5p sponge. **A** The localization of circHIPK3 was observed in cardiomyocytes by RNA FISH with a Cy3-labeled circHIPK3 probe. The nuclei were stained with DAPI. Scale bar = 20 μm. **B** The expression of miR-20b-5p in normal and H/R cardiomyocytes was detected by qRT-PCR. *P < 0.05 compared with the normal group. *n* = 9. **C** qRT-PCR analysis of miR-20b-5p expression in cardiomyocytes after cardiomyocytes were transfected with negative control (LV), circHIPK3 overexpression vector, or circHIPK3 siRNA (si-circHIPK3). **D** The relative luciferase activities were measured in 293T cells cotransfected with circHIPK3-WT or circHIPK3-Mut and miR-20b-5p mimics or miR-NC(MNC) by luciferase reporter assay. *P < 0.05 compared with the circHIPK3-Mut group. **E** FISH for circHIPK3 (red) and miR-20b-5p (green) was performed in cardiomyocytes. Scale bar = 20 μm.

Previous studies have reported that circRNA exerts its biological function by sponging miRNAs^19,20^. We determined whether circHIPK3 works via the same mechanism. An RNA FISH assay indicated that circHIPK3 was primarily expressed in the cytoplasm of cardiomyocytes (Fig. 3A), implying the potential posttranscriptional regulation of circHIPK3. Bioinformatics analysis through starBase (http://starbase.sysu.edu.cn/) revealed that miR-20b-5p contains binding sites for circHIPK3. To further investigate the regulatory relationship between miR-20b-5p and circHIPK3 expression levels in cardiomyocytes, We found that the expression of miR-20b-5p in cardiomyocytes decreased after H/R (Fig. 3B), while qRT-PCR results showed that circHIPK3 did not affect the expression of miR-20b-5p (Fig. 3C). Then, we conducted luciferase reporter assays and confirmed that ectopic expression of miR-20b-5p significantly inhibited the luciferase activity of circHIPK3-WT compared to that in the control group, but the luciferase activity of circHIPK3-Mut was not affected (Fig. 3D). Furthermore, FISH assay analysis showed that circHIPK3 and miR-20b-5p were colocalized in the cytoplasm of cardiomyocytes (Fig. 3E). Collectively, these data suggest that circHIPK3 can directly combine with miR-20b-5p and act as a sponge of miR-20b-5p.

### MiR-20b-5p inhibits autophagy and apoptosis of cardiomyocytes under H/R conditions

**Fig. 4:**
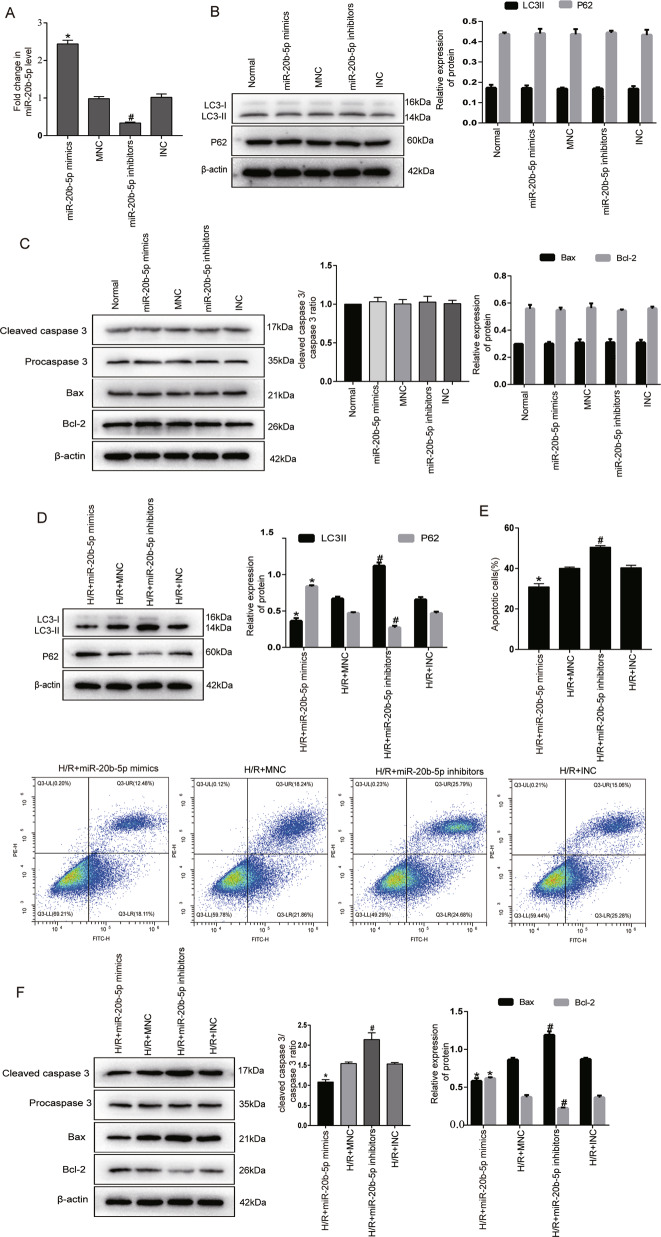
MiR-20b-5p inhibits autophagy and apoptosis of cardiomyocytes under H/R conditions. **A** Transfection efficacy of miR-20b-5p mimics and miR-20b-5p inhibitors in cardiomyocytes. **B** Western blot showed the effect of transfection of miR-20b-5p mimics and miR-20b-5p inhibitors on the expression of LC3II and P62 in normal cardiomyocytes. *n* = 3. **C** Western blot showed the effect of transfection of miR-20b-5p mimics and miR-20b-5p inhibitors on the expression of apoptosis-related proteins, including procaspase-3, cleaved caspase-3, Bax, and Bcl-2 in normal cardiomyocytes. *n* = 3. **D** Western blot showed the effects of miR-20b-5p mimics and miR-20b-5p inhibitor transfection on the expression of LC3II and P62 in cardiomyocytes under H/R conditions. *n* = 3. **E** Annexin V-FITC/PI flow cytometry was used to evaluate the effect of miR-20b-5p mimics and miR-20b-5p inhibitors on cardiomyocyte apoptosis under H/R conditions. *n* = 3. **F** Western blot analyzed the expression of apoptosis-related proteins, including procaspase-3, cleaved caspase-3, Bax, and Bcl-2 in cardiomyocytes under H/R conditions by miR-20b-5p mimics and miR-20b-5p inhibitors transfection. *n* = 3. *P < 0.05 compared with the mimics-NC (MNC) group. #*P* < 0.05 compared with the inhibitors-NC (INC) group.

Next, we explored the potential role of miRNA-20b-5p in regulating autophagy and apoptosis in cardiomyocytes. MiR-20b-5p mimics and miR-20b-5p inhibitors were transfected into cardiomyocytes. The transfection effects were confirmed by RT-qPCR (Fig. 4A). We found that transfection of miR-20b-5p mimics and inhibitors had no effect on the autophagy and apoptosis of normal cultured cardiomyocytes (Fig. 4B, C). Therefore, we investigated the effect of miR-20b-5p on autophagy and apoptosis of cardiomyocytes under H/R conditions. Transfection of miR-20b-5p mimics significantly reduced the ratio of LC3-II to LC3-I and increased p62 expression in cardiomyocytes under H/R conditions compared to the mimics-NC (MNC) group. In contrast, transfection of miR-20b-5p inhibitors increased the ratio of LC3-II to LC3- I and decreased p62 expression (Fig. 4D). Flow cytometry and western blotting showed that compared to the MNC group, miR-20b-5p mimics decreased apoptosis, decreased cleaved caspase-3 and Bax expression, and increased Bcl-2 expression in cardiomyocytes under H//R conditions. In contrast, miR-20b-5p inhibitors significantly increased apoptosis and proapoptotic protein expression and decreased the expression of the antiapoptotic protein Bcl-2 (Fig. 4E, F).

### CircHIPK3 regulated autophagy and apoptosis via the circHIPK3/miR-20b-5p/ATG7 axis

**Fig. 5:**
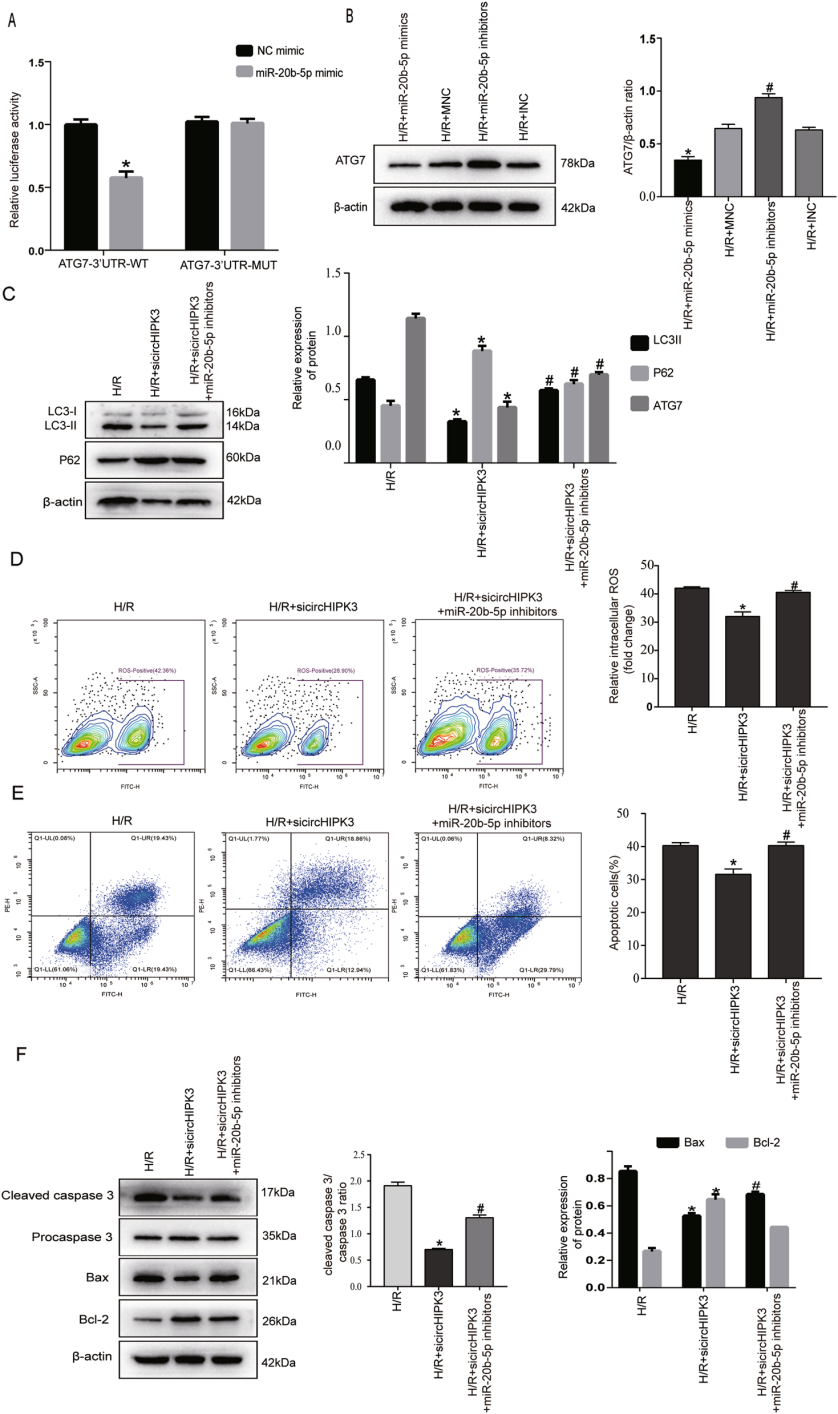
CircHIPK3 regulates autophagy and apoptosis via the CircHIPK3/miR-20b-5p/ATG7 axis. **A** Luciferase reporter assay showed that miR-20b-5p mimics directly binds to the 3′-UTR of ATG7 and inhibits luciferase activity. *P < 0.05 compared with the NC-mimics group. **B** ATG7 protein expression was detected by western blotting. *n* = 3. *P < 0.05 compared with the MNC group. #*P* < 0.05 compared with the INC group. **C** The protein expression levels of LC3-II, P62, and ATG7 were measured by western blotting. *n* = 3. **D** The intracellular ROS level was detected by flow cytometry. *n* = 3. **E** Annexin V-FITC/PI flow cytometry was used to evaluate apoptosis under different treatment conditions. *n* = 3. **F** Apoptosis-related proteins, including procaspase-3, cleaved caspase-3, Bax, and Bcl-2, were detected by western blotting. *n* = 3. *P < 0.05 compared with the H/R group. ^#^*P* < 0.05 compared with the H/R + sicircHIPK3 group.

Next, we used the online tools TargetScan and miRanda to predict the possible target genes of miR-20b-5p. One of the downstream genes, ATG7, aroused our interest. ATG7 is a ubiquitin E1-like ligase that activates ubiquitin-like proteins ATG12 and ATG8 and is correlated with LC3-II formation^21,22^. Therefore, we hypothesized that circHIPK3-mediated autophagy and apoptosis may be related to ATG7. To test whether miR-20b-5p directly targets ATG7, we performed luciferase reporter assays and designed a vector carrying the ATG7 3′-UTR. The data show that transfection of miR-20b-5p mimics reduced the luciferase activity of the wild-type ATG7 3′-UTR construct compared with that in the control group but did not affect the activity of the mutant 3′-UTR construct (Fig. 5A). Moreover, miR-20b-5p inhibited the expression of ATG7 at the protein level (Fig. 5B). These results demonstrated that ATG7 is a direct target of miR-20b-5p.

To further explore the relationship among circHIPK3, miR-20b-5p, and ATG7, cells were cotransfected with sicircHIPK3 and miR-20b-5p inhibitors. ATG7 protein expression, cell autophagy, and apoptosis were measured accordingly. Western blotting analysis showed that silencing circHIPK3 markedly decreased the expression of LC3-II, ATG7, cleaved caspase-3, and Bax and increased the expression of p62 and Bcl-2. Flow cytometry revealed that silencing circHIPK3 reduced ROS production and the number of apoptotic cells. Importantly, these effects could be reversed by miR-20b-5p inhibitors (Fig. 5C–F). In summary, these data demonstrated that circHIPK3 acts as a sponge for miR-20b-5p upregulates ATG7 expression and promotes cardiomyocyte autophagy and apoptosis induced by H/R injury.

## Discussion

In the current study, we found that autophagy activity was significantly enhanced and the expression of circHIPK3 was increased in the postischemic myocardium and in H/R-stimulated cardiomyocytes when compared with the control groups, indicating that the increase in circHIPK3 may be involved in the regulation of myocardial autophagy after I/R injury. Through gain-of-function and loss-of-function analyses of circHIPK3, we observed that overexpression of circHIPK3 promotes autophagy and apoptosis of H/R-stimulated cardiomyocytes while silencing circHIPK3 shows the opposite effect. Mechanistically, circHIPK3 directly sponges miR-20b-5p and upregulates the expression of the miR-20b-5p target gene ATG7, thereby promoting the autophagy and apoptosis of H/R-injured cardiomyocytes.

Autophagy and apoptosis are essential for maintaining the normal function of cardiomyocytes^23^. Autophagy and apoptosis are dysregulated in cardiovascular disease and regulate its pathogenesis^24,25^. Studies have shown that both abnormal autophagy and apoptosis are dysregulated in ischemic areas after myocardial infarction. In heart failure, excessive autophagy and apoptosis lead to further cardiomyocyte damage^26^. In myocardial infarction, enhancing the level of autophagy of cardiomyocytes significantly reduces the infarct size^27^. Whether autophagy is a damage factor or a protective factor remains to be further studied. However, there is increasing evidence that inhibiting autophagic cell death protects cardiomyocytes during I/R injury. During the I/R process, when the ischemic myocardial tissue restores the supply of nutrients and oxygen, a large amount of ROS is produced, which shows that autophagy is significantly increased. The excessive increase in autophagy levels is usually accompanied by increased autophagic and apoptotic death of cardiomyocytes.

In line with these studies, our current findings indicate that H/R injury induces cardiomyocyte autophagy and apoptosis. In this study, both circHIPK3 upregulation and excessive autophagy were observed in H/R-treated cardiomyocytes. Silencing circHIPK3 reduced autophagy and apoptosis induced by H/R injury compared to those in the control group. CircHIPK3 is a highly abundant circular RNA that mainly originates from the second exon of the HIPK3 gene^28^. circHIPK3 can bind multiple miRNAs, including miR-124 and miR-379^29^. In hepatocellular carcinoma, circHIPK3 can act as a sponge to absorb miR-124 and upregulate the downstream target genes IL6R and DXL2 through a competing endogenous RNA (ceRNA) mechanism, thereby promoting the proliferation of hepatoma cells. Researchers have shown that circHIPK3 promotes the proliferation of endothelial cells by competitively binding miR-30a-3p and boosting the expression of its downstream gene VEGF/WNT2/FZD4. This indicates that circHIPK3 could promote cell proliferation and migration and regulate vascular function. However, the potential involvement of circHIPK3 in cardiac autophagy remains unclear; therefore, we focused on revealing the function of circHIPK3 in cardiomyocytes under H/R conditions. Through bioinformatic prediction and experimental validation, we revealed that circHIPK3 can enhance H/R-induced cardiomyocyte autophagy and apoptosis by sponging miR-20b-5p. In general, miRNAs regulate gene expression by binding to the 3′-UTR of target mRNAs^30^. Our data revealed that miRNA-20b-5p was able to directly target the 3′-UTR of ATG7. ATG7 is an autophagy-related gene with ubiquitin E1-like activity that activates ubiquitin-like protein ATG12 during the extended phase of autophagy. ATG7 also participates in LC3-I activation and promotes the combination of LC3-I and phosphatidylethanolamine to form LC3-II. We found that ATG7 expression was positively correlated with circHIPK3 expression. However, miR-20b-5p reversed the regulatory effect of circHIPK3 on ATG7.

**Fig. 6:**
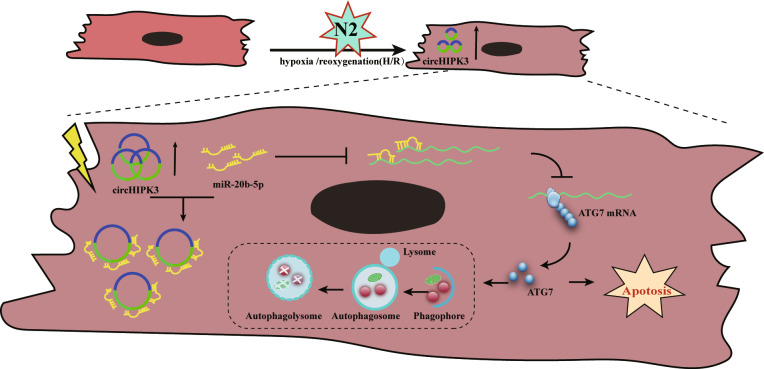
A schematic diagram of the circHIPK3/miR-20b-5p/ATG7 axis in cardiomyocytes. In H/R-stimulated cardiomyocytes, circHIPK3 sponges endogenous miR-20b-5p to sequester and inhibit the activity of miR-20b-5p, which leads to increased expression of ATG7, thereby regulating the autophagy and apoptosis of cardiomyocytes.

In conclusion, we found that circHIPK3 is significantly upregulated in H/R-stimulated cardiomyocytes compared to control cardiomyocytes and that this upregulation promotes cardiomyocyte autophagy and apoptosis. Mechanistically, circHIPK3 increases ATG7 expression by acting as a miR-20b-5p sponge (Fig. 6). In short, circHIPK3 mediates the process of autophagy and apoptosis by regulating the miR‐20b‐5p/ATG7 pathway. Therefore, circHIPK3 may become a potential therapeutic target for myocardial I/R injury.

## Materials and methods

### Animals

C57BL/6J (male and female, 1- to 3-day-old, 6- to 8-week-old) mice were purchased from Zunyi Medical University. All mice were divided into groups randomly. All experimental procedures were approved by the Institutional Animal Care and Use Committee of the University of Zunyi Medical University. The study was approved by the Medical Ethnics Committee of Zunyi Medical University (approval number: ZMUER2018-2-177).

### Cardiomyocyte culture

Neonatal mouse ventricular cardiomyocytes were isolated from 1- to 3-day-old C57BL/6J mice as previously described^12,13^. Briefly, neonatal mice were euthanized after performing heparinization for 5–10 min. Then, the chest was opened along the sternum, the heart was placed in a dish containing D-Hanks solution, and the atrium and connective tissue were quickly removed. The ventricles were minced into small pieces by eye scissors, and the heart was digested in 0.08% trypsin and 0.1% type II collagenase mixture at 37 °C for 10 min three or four times until the tissue block disappeared. The supernatant of each digestion was collected and resuspended in a complete medium containing 5% fetal calf serum. Cardiac fibroblasts and cardiomyocytes were separated according to different adhesion times. The cells were collected by centrifuging the supernatant, and the pellet was resuspended in a complete medium containing 10% FBS and 0.1 mmol/L 5-BrdU and incubated at 37 °C and 5% CO_2_ for 90 min. Then, the supernatant composed mainly of cardiomyocytes was collected and precipitated.

### Establishment of cellular models of H/R in vitro

To establish the H/R models, cardiomyocytes were cultured in low-glucose Dulbecco’s Modified Eagle Medium complete medium without FBS in a Galaxy® 48 R incubator (Eppendorf/Galaxy Corporation, USA) with 1% O_2_, 5% CO_2_, and 94% N_2_ for 6 h (hypoxic conditions). The cells were then moved to normal conditions containing 95% air and 5% CO_2_ for 4 h.

### Transmission electron microscopy (TEM) analysis

For TEM analysis, cardiomyocytes were cultured in 6-well plates under similar treatments mentioned above. Then, the cell was washed in 0.1 M phosphate-buffered saline (PBS) and fixed in 2.5% glutaraldehyde at 4 °C for 2–4 h. Cells were collected and fixed in 1% osmium acid at room temperature for 2 h. Thereafter, the specimens were dehydrated with ascending concentrations of alcohols (from 50 to 100%) and then embedded in Spurr’s resin. Ultrathin (60–80 nm) sections were cut and placed on copper mesh grids. The sections were then double-stained with 1% uranyl acetate and lead citrate. Ultrastructural analysis of the heart was also performed. The samples were examined under a transmission electron microscope.

### Flow cytometry analysis of apoptosis

We used annexin V-fluorescein isothiocyanate (FITC)/propidium iodide (PI) staining (Solarbio, China) to detect phosphotidylserine exposure to the outer surface of the cell membrane according to the manufacturer’s instructions. Briefly, cells were cultured in a 6-well plate, and after the required treatment, cells were trypsinized and resuspended in binding buffer at a density of 1 × 106 cells/ml. Then, the cells were incubated in annexin V and propidium iodide, and the specimens were placed in the dark for 15 min and analyzed via a FACS Calibur flow cytometer (BD Biosciences, USA).

### Flow cytometry analysis of intracellular reactive oxygen species (ROS)

Intracellular ROS production was detected by 2′,7′-dichlorofluorescein diacetate (DCFH-DA) staining (Sigma, USA) according to the manufacturer’s instructions. Briefly, cells were incubated with DCFH-DA probe for 30 min at 37 °C, washed three times with PBS, trypsinized, and centrifuged. The fluorescence intensity was analyzed by flow cytometry.

### Real-time qPCR

Total RNA was isolated from cells using TRIzol reagent (Life Technologies, Carlsbad, CA). Complementary deoxyribonucleic acid was synthesized from total RNA by PrimeScript RT Master Mix (Takara, Dalian, China), and qPCR was performed using TB Green Premix Ex Taq II (Takara). The amount of miRNA was detected by stem-loop RT-PCR. GAPDH or U6 were used as internal standards. The relative expression of genes was calculated using the 2^−ΔΔCt^ method.

### RNA fluorescence in situ hybridization (RNA FISH)

Cy3-labeled circHIPK3 and FITC-labeled miR-20b-5p probes were used to observe the colocalization of circHIPK3 and miR-20b-5p in cells. The FISH assay was conducted using the Fluorescent In Situ Hybridization Kit (Geneseed, Guangzhou, China) according to the manufacturer’s protocols. Nuclei were counterstained with 4,6-diamidino-2-phenylindole (DAPI, Beyotime, China), and fluorescence images were acquired by using a fluorescence microscope.

### Luciferase reporter assay

Sequences of circHIPK3/ATG7 containing wild-type or mutant miR-20b-5p binding sites were synthesized and inserted into the pmirGLO luciferase vector (GeneCreate, Wuhan, China). HEK293T cells were seeded into 96-well plates at a density of 5 × 103 cells per well and incubated in an incubator for 24 h, then cotransfected with reporter plasmids and miR-20b-5p mimics or negative control (NC) using Lipofectamine 2000 (Invitrogen). After 48 h, luciferase activity was detected using the Dual-Luciferase Assay System (Promega, Madison, WI).

### Cell transfection

CircHIPK3-overexpressing lentivirus (LV-circHIPK3), empty vector lentivirus, and small interfering RNAs targeting circHIPK3 (LV-sicircHIPK3) were synthesized by HanBio (Shanghai, China). MiR-20b-5p mimic, miR-20b-5p inhibitor, and negative controls were synthesized by Ribobio (Guangzhou, China). Cells were transfected using Lipofectamine 2000 (Invitrogen, Carlsbad, CA, USA) according to the manufacturer’s instructions.

### Western blot analysis

Total protein was extracted from cells using RIPA buffer (Biosharp, China), and then the protein concentration was quantified using a bicinchoninic (BCA) protein assay kit (Beyotime, China). Proteins were separated using 10% SDS-polyacrylamide gel electrophoresis (SDS-PAGE) and transferred to PVDF membranes (Millipore, Billerica, MA, USA). The membranes were blocked in 5% nonfat milk for 1 h and incubated with primary antibodies overnight at 4 °C. The following primary antibodies were used: rabbit anti-LC3 (Cell Signaling Technology, 12741), rabbit anti-Beclin1 (Cell Signaling Technology, 3495), rabbit anti-ATG7 (Cell Signaling Technology, 8558), rabbit anti-P662 (Cell Signaling Technology, 16177), rabbit anti-cleaved caspase 3 (Cell Signaling Technology, 9664), rabbit anti-caspase 3 (Cell Signaling Technology, 14220), rabbit anti-Bax (BOSTER, A00183), rabbit anti-Bcl-2 (BOSTER, A00040-2), rabbit anti-β-actin (BOSTER, BA2305). Afterward, the membranes were incubated with secondary antibody at room temperature for 1 h. An ECL kit (Beyotime Biotechnology, Shanghai, China) was used to detect the bands of the western blots.

### Animal I/R injury model

Mice underwent inhalational induction of anesthesia with 2% isoflurane, and the left anterior descending artery was sutured with 5–0 sutures for 30 min and then perfused for 2 h to induce a myocardial I/R injury model in vivo. The sham operation group underwent the same procedure, except the suture was not tied. The hearts were collected for circHIPK3 detection and electron microscopic observation.

### Statistical analysis

The analysis was performed using the SPSS 21.0 statistical software package (IBM, Armonk, NY, USA). The data were normally distributed and are expressed as the mean ± SD. One-way analysis of variance (ANOVA) was used to compare multiple groups. In addition, *P* values less than 0.05 were considered statistically significant.

## Data Availability

The data used to support the findings of this study are included within the article.
